# P-1718. Use of Collaboration to Harmonize Antimicrobial Registry Measure (CHARM) to Assess Antibiotic Prescribing in Dental Practices

**DOI:** 10.1093/ofid/ofae631.1883

**Published:** 2025-01-29

**Authors:** Cameron Smith, Minji Sohn, Benjamin Pontefract, Kushal Dahal, Michael Klepser, Helen Mishreky

**Affiliations:** Ferris State University, Grand Rapids, Michigan; Ferris State University, Grand Rapids, Michigan; Ferris State University, Grand Rapids, Michigan; Ferris State University, Grand Rapids, Michigan; Ferris State University, Grand Rapids, Michigan; Ferris State University, Grand Rapids, Michigan

## Abstract

**Background:**

Dentists account for 10% of all antibiotic prescriptions in the United States. Limited data exist describing antibiotic use in dental practices; however, it appears that many antibiotics may not be warranted or consistent with treatment guidelines.1,2,3 Of particular concern is the unwarranted use of clindamycin. No standardized methods exist for gathering data and assessing the concordance of use related to antibiotic use in dental practices.

**Objectives:**

Describe patterns of antibiotic prescribing in dental clinics within the CHARM network. Identify areas for stewardship intervention within dental practices. top 5 antibiotic prescription in dental clinics

**Figure:**
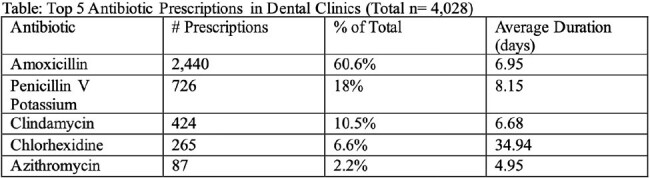

name of antibiotic, the number of prescriptions, percentages, and average duration

**Methods:**

Dental practices were identified from 5 dental clinics that participate in CHARM. The CHARM database is a registry containing data collected from the electronic medical records of partnering health systems about outpatient antibiotic prescribing. Episodes of antibiotic prescribing were identified in dental clinics and extracted between January 2022 to June 2023. Data collected included the ICD-10 code, antibiotic, and regimen used. When a diagnosis was provided, the appropriateness of antibiotic selection was assessed in concordance with guidelines. The most common antibiotics and diagnoses were identified.

**Results:**

4,028 antibiotic prescriptions were written by 42 prescribers in 5 dental clinics. The most prescribed antibiotics were amoxicillin (n=2,400; 59.6%), penicillin (n=700;17.3%), and clindamycin (n=400;9.9%). The average length of therapy was 7.0, 8.2, and 6.9 days for amoxicillin, penicillin, and clindamycin, respectively. ICD-10 codes were recorded for 1,722 (43%) prescriptions. No indication/diagnosis was provided for 2,278 (57%) antibiotic prescriptions. While many prescriptions were not linked to any diagnosis (n=2,300; 57.1%), the most common diagnosis codes were general dental exam (n=1,400; 34.8%) and general dental infection (n=300; 7.4%).

**Conclusion:**

For visits where the dentist prescribed antibiotics, clindamycin was prescribed about 10% of the time. Clindamycin is no longer recommended for use in most procedures that dentists manage.^4^ Reducing clindamycin use in dental practices represents a stewardship opportunity in dental practices.

**Disclosures:**

**Minji Sohn, PhD**, Emergent BioSolutions, Inc.: Grant/Research Support

